# Curcumin inhibits endometriosis endometrial cells by reducing estradiol production 

**Published:** 2013-05

**Authors:** Ying Zhang, Hong Cao, Zheng Yu, Hai-Ying Peng, Chang-jun Zhang

**Affiliations:** 1 *Reproductive Medicine Center, Renmin Hospital, Hubei University of Medicine, Shiyan, Hubei, P.R. China.*; 2 *Department of Orthopedic Surgery, Renmin Hospital, Hubei University of Medicine, Shiyan, Hubei, P.R. China.*

**Keywords:** *Curcumin*, *Endometriosis*, *Estradiol*, *Stromal cell*, *Epithelial cell*

## Abstract

**Background: **Endometriosis is a complex estrogen-dependent disease that is deﬁned as the presence of endometrial gland and stroma outside the uterine cavity. Although the exact mechanism for the development of endometriosis remains unclear, there is a large body of research data and circumstantial evidence that suggests a crucial role of estrogen in the establishment and maintenance of this disease.

**Objective:** This study is an attempt to assess the effect of curcumin on inhibiting endometriosis endometrial cells and to investigate whether such an effect is mediated by reducing estradiol production.

**Materials and Methods: **Endometriotic stromal cells, normal endometrial stromal cells, endometriotic epithelial cells and normal endometrial epithelial cells were isolated and cultured. E_2_ value of cells and the effect of curcumin on cell proliferation were evaluated. Finally, effect of curcumin on E_2_ assay was detected.

**Results:** Electrochemiluminescence immunoassay results showed that E_2_ value of endometriotic epithelial cells was higher than the endometriotic stromal cells (p=0.037), while the expression of E_2_ in normal endometrial stromal and epithelial cells was extremely low. WST-8 result showed, compared with endometrial stromal cells, ectopic endometriotic stromal cells had a higher growth rate. After intervene with curcumin (10μmol/L, 30μmol/L and 50μmol/L) for 0-96h, the number of endometriotic stromal cells was reduced and cells growth slowed, compared with 0μmol/L group. Compared with 0μmol/L group, E_2_ level was lower after treatment with curcumin, especially in 30μmol/L and 50μmol/L group.

**Conclusion:** In summary, in this study we found that E_2_ is important in ectopic endometrium, and epithelial cell is in dominant position with E_2_ secretion. Curcumin was able to suppress the proliferation of endometrial cells by reducing the E_2_ value.

## Introduction

Endometriosis is defined as the presence of endometrial tissue in an abnormal location. It is an enigmatic, debilitating disease which affects as many as 15% of all women of reproductive age, and is characterized by pelvic pain and infertility (1). Similar to other chronic diseases, endometriosis is inherited in a polygenic manner and has a complex and multifactorial etiology. 

Although the exact mechanism for the development of endometriosis remains unclear, there is a large body of research data and circumstantial evidence that suggests a crucial role of estrogen in the establishment and maintenance of this disease (2, 3). Estradiol (E_2_) is an important promoter of the growth of both eutopic and ectopic endometrium. The primary source of E_2_ is the ovary, and E_2_ has been recently found to be an effective regulator of endometriosis (4). 

**Figure 1:**
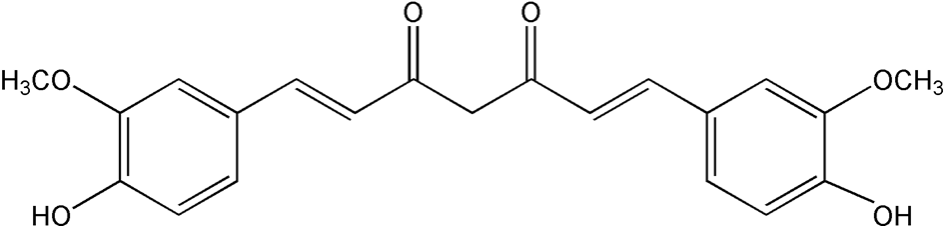
Chemical structural formula of curcumin (C_21_H_20_O_6_

Curcumin is a naturally occurring phytochemical and an extract of turmeric. Curcumin has potent anti-inflammatory, antioxidant, antiangiogenic, anti-neoplastic properties and is used as a therapeutic agent in Indian and Chinese medicine (5). Extensive in vitro and in vivo data have paved the way for curcumin to become the subject of clinical trials. Early-phase trials have ascertained pharmacological properties and consistently demonstrate it to be safe and well tolerated (6, 7). In this study, curcumin (molecular weight 368.4, purity 99%) was purchased from Fluka Chemie GmbH (USA). The chemical structural formula of curcumin is shown in figure 1. 

Neither the etiology nor the pathogenesis of endometriosis is fully understood. Higher levels of E_2_ have been reported in menstrual blood of endometriosis patients, in comparison with healthy women, suggesting that in these endometriosis patients E_2_ is formed locally in the endometrium. In addition, the successful treatment of several cases of endometriosis using aromatase inhibitors, which prevent the local formation of estrogens, further supports the estrogen dependency of endometriosis. Moreover, several case reports of histological endometriosis in elderly men undergoing high-dose estrogen therapy for prostate cancer also support this estrogen dependency (8).

Current treatment regimens manage the disease by inducing a hypoestrogenic state (9). In preliminary work, we found that curcumin has the ability of inhibiting endometriosis in vivo (10). In order to study the physiopathology of endometriosis and clarify the relationship between E_2_ and curcumin, we performed in vitro study described in this communication. Four different endometrial cells were cultured: endometriotic stromal cells, normal endometrial stromal cells, endometriotic epithelial cells and normal endometrial epithelial cells. Curcumin as new therapeutic reagent was then tested, hoping for finding a new way to treat endometriosis.

## Materials and methods

As mentioned in introduction, in preliminary work, we found that curcumin has the ability of inhibiting endometriosis in vivo, so in order to study the physiopathology of endometriosis and clarify the relationship between E_2_ and curcumin, we performed this in vitro study. 

Eutopic endometrial cells with endometriosis were obtained from premenopausal patients who had undergone hysterectomies for endometriosis (n=8, aged 24-45 yr). The patients with endometriosis were confirmed by laparoscopic diagnosis, menstrual regularity, cycle 28-32 days, excluding other endocrine, immunological or metabolic diseases. The patients did not receive hormone therapy within six months prior to surgery. Chocolate cyst was scraped by hysteroscopy. 

A portion of endometrial tissue was taken and fixed in 10% neutral formalin and sent to pathology testing immediately, malignant lesions were discarded. The remaining specimens have been frozen into DMEM sterile tube (contain penicillin and streptomycin 10μl each), and immediately sent back to the lab. 

Normal endometrial cells were obtained from premenopausal patients who had undergone hysteroscopy for infertility and had no evidence of endometriosis (n=9, aged 29-43 yr). The endometrium was scraping in the hysteroscopic examination. A portion of endometrial tissue was taken and fixed, endometrial tissue was confirmed by histologically and excluded endometriosis, malignant lesions. This study is supported by Hubei Provincial Department of Education (No.Q20132108).

The ages of the patients were statistically similar between the two groups. All patients were free of any hormonal treatments before the operation. This study was approved by the ethics committee of Hubei University of Medicine and written informed consent was obtained from all subjects. All the specimens were diagnosed as being in the mid. to late secretory phase using a standard histological examination of endometrial tissues. This study was approved by the Reproductive Medicine Center, Renmin Hospital, Hubei University of Medicine, Shiyan, China.

Curcumin (molecular weight 368.4, purity 99%) was purchased from Fluka Chemie GmbH (USA). It was dissolved in DMEM solution, and sterilized through 0.22μm membrane filtration. Before use, it was diluted to the desired concentrations as following: 10μmol/L, 30μmol/L and 50μmol/L with DMEM containing 20% fetal calf serum (FBS).

Endometriotic stromal cells, normal endometrial stromal cells, endometriotic epithelial cells and normal endometrial epithelial cells were isolated by digesting the endometrial tissue fragments with 0.5% collagenase. Isolated cells were cultured in DMEM supplemented with 1% penicillin/ streptomycin (Sigma-Aldrich, St Louis, MO, USA), and 10% charcoal-stripped heat-inactivated FBS (Life Technologies, Inc.-BRL) in a humidified atmosphere of 5% CO_2_ and 95% air at 37^o^C. Cells isolated from each individual patient were used for one experiment at a time. DMEM was checked by an experienced person.

For E_2_ assay, endometriotic stromal cells, normal endometrial stromal cells, endometriotic epithelial cells and normal endometrial epithelial cells were plated in 6-well plates at a density of 6×10^5^ cells/ml. After incubation for 24 hours, the cells were centrifuged at 700rpm for 5mins. Supernatant was collected. E_2_ concentration in the supernatant of each group was measured by electrochemiluminescence immunoassay method.

The effect of curcumin on cell proliferation was evaluated. Endometriotic cells and normal endometrial cells were plated in flat-bottomed 96-well plates (Corning, NY, USA) (6×10^5^ cells per well). After incubation for 24 hours, the culture medium was replaced to medium containing curcumin at concentration of 10μM, 30μM or 50μM. 10μl WST-8 were added into the culture medium into each well at various time points ranging from 0h, 24h, 48h, 72h, to 96h. Four hours after the addition of WST-8, medium then was decanted, and the absorbance was determined at 450 nm using an enzyme-linked immunosorbent assay (ELISA) reader (BIO-TEK Instruments, Winooski, VT, USA). Finally, effect of curcumin on E_2_ assay was detected. Cells were plated in 6-well plates at 6×10^5^ cells/mL and treated with curcumin at concentrations of 10μM, 30μM or 50μM. 200ul cell supernatant was collected at five time points ranging from 0h, 24h, 48h, 72h, to 96h. E_2_ concentration was detected as mentioned above.

**Statistical analysis**

Statistical analysis was performed using the statistical package for the social science (SPSS software version 12.0 for windows, Chicago, IL), a Student's t-test was used to detect significant differences (p<0.05) of the all variables between the groups.

## Results

**Figure 2:**
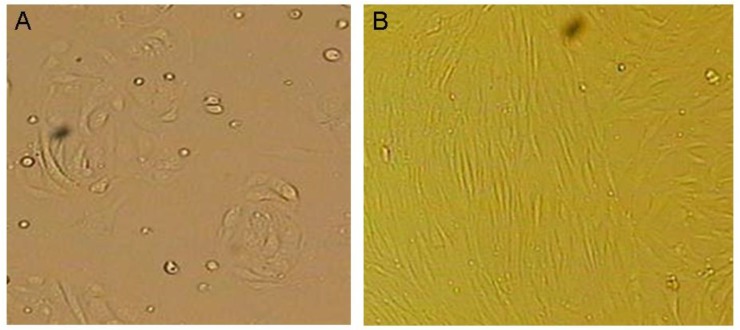
Epithelial cells were polygonal, with thin and transparent cytoplasm and center located round nuclear. After 3-4 days culture, cells developed the appearance of decidual cell-like transformation with larger size. The cytoplasms were large and bright and the size of nucleus significantly increased. Cells were arranged in spiral-like or dough pattern with gradually outward derivative, and showing filamentous connection (A). Stromal cells had fibroblast morphology, spindle-shaped, abundant cytoplasm, oval-shaped nuclear. They were easy to passage. After long-term culture, these cells were arranged in parallel bundles, with dense piles of regional aggregation (B).

Epithelial cells were polygonal, with thin and transparent cytoplasm and center located round nuclear. After 3-4 days culture, cells developed the appearance of decidual cell-like transformation with larger size. The cytoplasms were large and bright and the size of nucleus significantly increased. Cells were arranged in spiral-like or dough pattern with gradually outward derivative, and showing filamentous connection (Figure 2A). Stromal cells had fibroblast morphology, spindle-shaped, abundant cytoplasm, and oval-shaped nuclear. 

**Figure 3:**
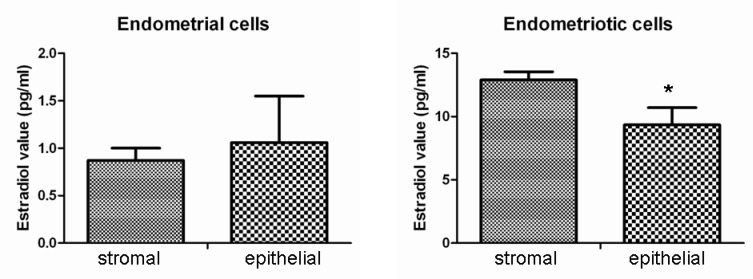
Electrochemiluminescence immunoassay results showed that E_2_ value of endometriotic epithelial cells was 12.90±0.6pg/ml, higher than that of the endometriotic stromal cells which is 9.34±1.37 pg/ml. There was a significant difference between them (p=0.037); E_2_ values of normal endometrial stromal cells and normal endometrial epithelial cells are extremely low, almost close to the minimum value of detection

They were easy to passage. After long-term culture, these cells were arranged in parallel bundles, with dense piles of regional aggregation (Figure 2B). Morphology between endometriotic stromal cells and normal endometrial stromal cells, endometriotic epithelial cells and normal endometrial epithelial cells were similar. No significant difference was observed. Electrochemiluminescence immunoassay results showed that after isolation and purification, E_2_ value of endometriotic epithelial cells was 12.90±0.6 pg/ml, higher than that of endometriotic stromal cells (9.34±1.37 pg/ml). E_2_ value of normal endometrial stromal cells and normal endometrial epithelial cells were extremely low, almost close to the minimum value of detection (Figure 3).

Epithelial cells, is the primary cell type of the first subculture. When passaged to the second generation, the basic epithelial form of morphology could not be identified. Stromal cells were fewer in primary culture, but after repeated passages, their cellular growth and vitality increased. They became the main subcultured cells. Based on the above observation, the stromal cells (including endometriotic and normal endometrial) were selected for the following experiments. 

**Figure 4:**
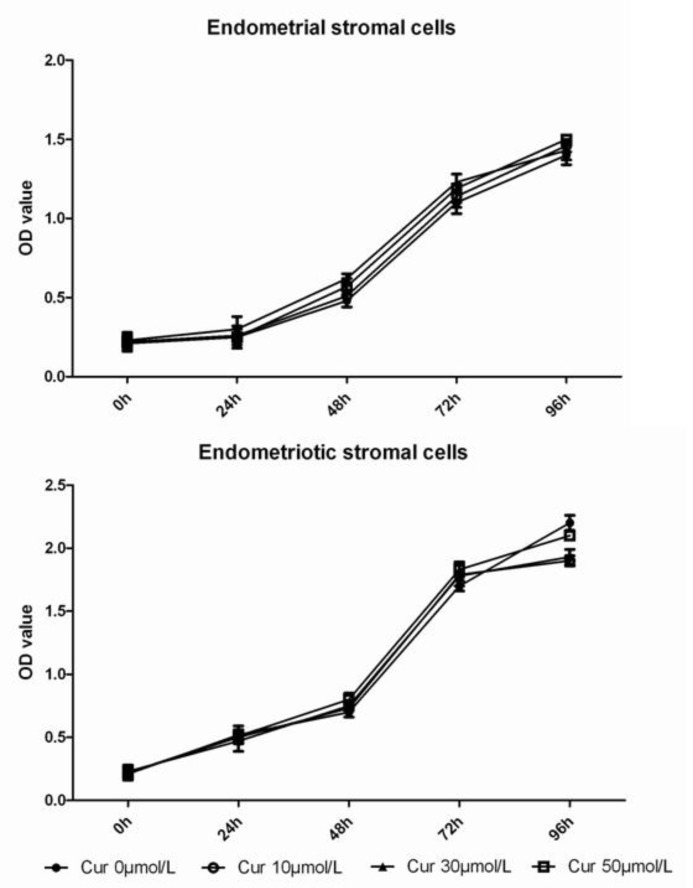
Compared with endometrial stromal cells, ectopic endometrial stromal cells with the corresponding time points had a higher cell number. After intervene with curcumin (10μmol/L, 30μmol/L and 50μmol/L) for 96h, the number of endometriotic stromal cells was reduced, compared with 0μmol/L group

Compared with endometrial stromal cells, ectopic endometrial stromal cells in vitro had a higher growth rate and cell number shown by WST-8 assay (Figure 4A). After intervene with curcumin (10μmol/L, 30μmol/L and 50μmol/L) for 96h, the number of endometriotic stromal cells was reduced and cell growth slowed, compared with 0μmol/L group (Figure 4B). The morphology of these endometrial stromal cells did not changed significantly. 

**Figure 5:**
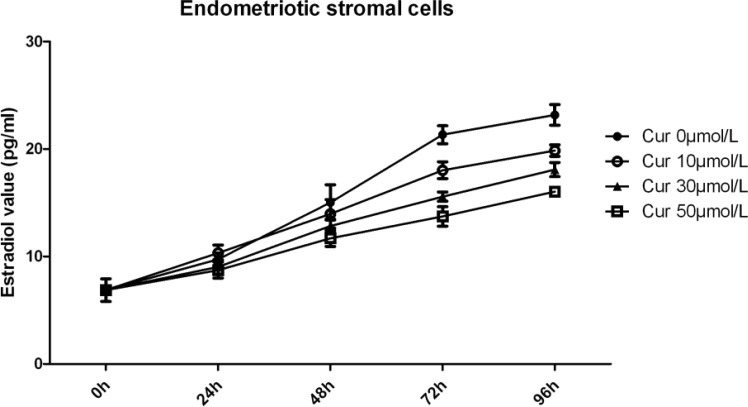
E_2_ level of endometriotic stromal cells was increased with time. Compared with previous time point, E_2_ level was increased by 33.18% (24hr), 57.28% (48hr), 38.80% (72hr) and 12.45% (96hr). Compared with 0μmol/L group, E_2_ level was lower after intervene with curcumin, especially in 30μmol/L and 50μmol/L groups (Cur 10μmol/L vs. Cur 0μmol/L p=0.094, Cur 30μmol/L vs. Cur 0μmol/L p=0.045, Cur 50μmol/L vs. Cur 0μmol/L p=0.026

E_2_ level of endometriotic stromal cells was increased with time. Compared with previous time points, E_2_ level was increased by 33.18% (24hr), 57.28% (48hr), 38.80% (72hr) and 12.45% (96hr). Compared with 0μmol/L group, E_2_ level was lower after intervene with curcumin, especially in 30μmol/L and 50μmol/L groups (Cur 10μmol/L vs. Cur 0μmol/L p=0.094, Cur 30μmol/L vs. Cur 0μmol/L p=0.045, Cur 50μmol/L vs. Cur 0μmol/L p=0.026) (Figure5).

## Discussion

Endometriosis is a complex estrogen-dependent disease that is deﬁned as the presence of endometrial glands and stroma outside the uterine cavity (11, 12). Endometriosis primarily affects women of reproductive age. It is occasionally diagnosed in postmenopausal women, usually in those with relatively high estrogen levels, or those who are treated with estrogen-replacement therapy. Higher levels of E_2_ have been reported in menstrual blood of endometriosis patients, in comparison with healthy women, suggesting that in these endometriosis patients E_2_ is formed locally in the endometrium (13).

In addition, the successful treatment of several cases of endometriosis using aromatase inhibitors, which prevent the local formation of estrogens, further supports the estrogen dependency of endometriosis (14-16). In our study, E_2_ values of normal endometrial stromal cells and normal endometrial epithelial cells are extremely low, almost close to the minimum value of detection. In contrast, the E_2_ values of endometriotic epithelial and stromal cells are much higher, which confirmed that E_2_ is an effective regulator of endometriosis. 

Endometrium is composed by epithelial cells and stromal cells. The importance of endometrial epithelial-stroma interactions in the acquisition of epithelial receptivity has been documented in previous studies (17). Lessey *et al* demonstrated that progesterone, acting through endometrial stromal progesterone receptors (PRs), induces HB-EGF release from stromal cells (18). HB-EGF acts in a paracrine way on epithelial cells by increasing the expression of integrin beta3 and thus increases epithelial receptivity. In this study, primary epithelial cell cultures retain their original characteristics over the ﬁrst to second passages and start to change thereafter. 

That may be due to the lack of material and signal exchange between isolated and cultured epithelial cells and stromal cells in in vitro system. Epithelial cells lacking the necessary material and signal control, had slower growth rate or committed cell death. Therefore, we can speculate that stromal cells play an important supporting role. Beliard *et al* indicated that the success of endometrial cell implantation is dependent on the cooperativeness between stromal and epithelial endometrial cells, as well as on the endocrine environment of endometrial cells, but not that of recipient animals (19). 

Their results emphasized the role of both endometrial cell types for ectopic implantation. Another interesting finding is that E_2_ value of endometriotic epithelial cells was higher than that of the endometriotic stromal cells, suggesting that epithelial cell is in dominant position with E_2_ secretion in endometrium. Considering epithelial cells is the primary cell type of the first subculture, maybe E_2_ is the key factor in driving endometriosis. 

Curcumin is a major component of turmeric powder extracted from the rhizome of the plant Curcuma longa found in South and Southeast tropical Asia. Curcumin exhibits great promise as a therapeutic agent, and is currently in clinical trials for treating a variety of conditions (20-22). This study is an attempt to assess the effect of curcumin on inhibiting endometriosis endometrial cells and to study whether the effect is mediated by reducing estradiol production. Our ﬁndings reveal that curcumin was able to suppress the proliferation of endometrial cells by reducing E_2_ level. Bachmeier *et al *demonstrated estrogenic effects of putative phytoestrogens at physiological concentrations and showed estrogenic effects of curcumin (23). 

The activation of E_2_ genes by curcumin most probably is due to an estrogen receptor α mediated estrogen-like effect. Singh and Singh also showed that curcumin was able to counteract the proliferative response of E_2_, and induce apoptosis (24). In summary, in this in vitro study we found that E_2 _is important in ectopic endometrium. Epithelial cell is in dominant position with E_2_ secretion of endometrial cells. Curcumin was able to inhibit the proliferation of endometrial cells by reducing the E_2_ value which may be developed into a new way to treat endometriosis. 
